# Associations between changes in behavioral difficulties and levels of problematic smartphone use in adolescents over a 1-year period

**DOI:** 10.1007/s00787-021-01874-8

**Published:** 2021-09-21

**Authors:** Tanja Poulain, Mandy Vogel, Tobias Kliesener, Wieland Kiess

**Affiliations:** 1 LIFE Leipzig Research Center for Civilization Diseases, Leipzig University, Philipp-Rosenthal-Strasse 27, 04103 Leipzig, Germany; 2 Department of Women and Child Health, University Hospital for Children and Adolescents and Center for Pediatric Research, Leipzig University, Liebigstrasse 20a, 04103 Leipzig, Germany

## Abstract

**Objectives:**

The present longitudinal study investigates associations between changes in externalizing and internalizing behavioral difficulties and changes in problematic smartphone usage within the same 1-year period in healthy adolescents.

**Methods:**

The project is part of the LIFE Child cohort study conducted in Leipzig, Germany. Ten- to 16-year-old adolescents (*n* = 363) provided information on behavioral difficulties [Strengths and Difficulties Questionnaire (SDQ)], the duration of daily smartphone use, and symptoms of smartphone addiction [Smartphone Addiction Proneness Scale (SAPS)] at two consecutive study visits, t1 and t2 (1 year after t1). In the first of two analysis phases, we applied linear regression analyses to assess cross-sectional associations between externalizing and internalizing behavioral difficulties and the duration of smartphone use and symptoms of smartphone addiction (at t1 and t2). In the second, we assessed associations between the changes measured in these variables over the period of a year. All associations were adjusted for age, sex, and soci-economic status.

**Results:**

Children who reported prolonged periods of smartphone use or more symptoms of smartphone addiction exhibited significantly higher levels of externalizing and internalizing behavioral difficulties at t1 and t2. Further, children who increased their usage or developed addiction symptoms between t1 and t2 also developed more externalizing behavioral difficulties. We found the same tendencies in regard to internalizing behavioral difficulties, although the associations did not reach statistical significance.

**Conclusions:**

The present findings suggest that externalizing behavioral difficulties and problematic smartphone use are mutually dependent in the long term.

## Introduction

The use of smartphones among children and adolescents has increased dramatically in recent years [1]. Even though a smartphone can simplify or enrich certain aspects of a young person’s life, excessive use may bring the risk of behavioral addiction [2]. According to the DSM-V [3], behavioral addiction (e.g., related to excessive internet use) is characterized by a dominance of a behavior in daily life, withdrawal symptoms, tolerance, loss of control, loss of interest in other activities, continued use despite psychosocial problems, the deception of family or friends, relief of negative mood through the behavior, and loss or missed opportunities in significant areas of life. Whether or not smartphone addiction qualifies as an addiction is a matter of debate. Experts have suggested the term “problematic smartphone use” to describe excessive use with negative effects on everyday functioning [2, 4].

Problematic smartphone use has been related to lower school success, dysfunctional relationships with parents, lower self-control, lower self-esteem [2], and more symptoms of internalizing difficulties, e.g., depression or anxiety [5]. Regarding associations with externalizing difficulties, previous study findings are mixed [6, 7]. Given that previous studies have taken a cross-sectional approach, they do not enable conclusions on long-term relationships or the direction of any effects. The present study aims to fill this research gap. With this in mind, we investigated associations between change (magnitude of increase or decrease) in problematic smartphone use within a period of 1 year and corresponding changes in internalizing and externalizing difficulties in the same time period.

## Methods

Data were collected between 2018 and 2020 as part of the LIFE Child cohort study conducted in Leipzig, Germany [8]. LIFE Child is a cohort study investigating development in healthy children and adolescents. Eligible children (not suffering from chromosomal or syndromal disease) have been recruited since 2011 between the age of 3 and 16 years via advertisement at public health centers, schools, and in the media, and by worth of mouth. All participants who owned a smartphone and completed all relevant questionnaires at two consecutive study visits (t1 and t2), approximately 12 months apart, were included in the present project.

Problematic smartphone use was assessed by symptoms of smartphone addiction and the duration of daily use. Symptoms of smartphone addiction were assessed using a German translation [9] of the Smartphone Addiction Proneness Scale (SAPS) [10]. The responses to the 15 items were combined to produce a total addiction score ranging from 15 to 60, with higher scores indicating a higher proneness to smartphone addiction. Cronbach’s alpha for this scale was 0.84. The duration of daily smartphone use was assessed using a media use questionnaire designed by the authors. Four questions on the participants’ daily smartphone use (online-weekday, online-weekend, offline-weekday, offline-weekend) were analyzed. For each question, the participants were asked to choose the most appropriate of five response options (never, approximately 30 min, 1–2 h, 3–4 h, > 4 h). The responses were converted to hours of daily use (0, 0.5, 1.5, 3.5, and 5). Finally, the responses to the four separate questions were combined to create the new variable “duration of daily smartphone use” (((online-weekday + offline-weekday) × 5) + ((online-weekend + offline-weekend) × 2)/7).

Behavioral difficulties were assessed using the Strengths and Difficulties Questionnaire (SDQ) [11]. Scores on the hyperactivity/inattention and conduct problems scales were combined to produce an “externalizing difficulties” score, and scores on the emotional problems and peer-relationship problems scales were combined in an “internalizing difficulties” score. Each combined score ranges from 0 to 20, with a higher score indicating greater difficulties. Cronbach’s alpha was 0.71 for both scales. The socio-economic status (SES) of study participants was measured using a composite SES score (adapted from [12]) combining information on the education, occupation, and equivalent household income of the participants’ parents. This score ranges from 3 to 21, with a higher score indicating higher SES. Based on cut-offs reported in a representative German study, the participants’ SES can be categorized as either low, middle or high [12].

The analysis process comprised two phases. For the first, we applied linear regression analyses to assess cross-sectional associations between externalizing and internalizing behavioral difficulties (as dependent variables) and duration of daily smartphone use and symptoms of smartphone addiction (as independent variables) at both t1 and t2. For the second, we assessed longitudinal associations between changes in externalizing or internalizing difficulties between t1 and t2 (as outcomes) and changes in daily smartphone use and symptoms of smartphone addiction (as predictors) using linear regression analyses. Association strength was indicated in each case by non-standardized regression coefficients. All associations were adjusted for the potential confounders age (in years), sex, and SES (score).

## Results

The final sample comprised 363 healthy children and adolescents [186 (51%) boys] aged 10–16 years (mean age at t1 = 13.4 years, sd = 1.63). The majority of participants’ parents (55%) had high SES, 38% middle SES, and 7% low SES. While, on average, the internalizing difficulties score increased between t1 (mean = 4.35, sd = 3.19) and t2 (mean = 4.68, sd = 3.34), the externalizing difficulties score showed a slight decrease [mean at t1 = 5.19 (sd = 2.97), mean at t2 = 5.09 (sd = 3.05)]. Regarding problematic smartphone use, both the duration of daily use [mean at t1 = 2.76 h (sd = 1.86), mean at t2 = 2.93 h (sd = 1.81)] and the degree of addiction [mean at t1 = 26.90 (sd = 6.47), mean at t2 = 27.68 (sd = 6.82)] increased between t1 and t2.

**Fig. 1:**
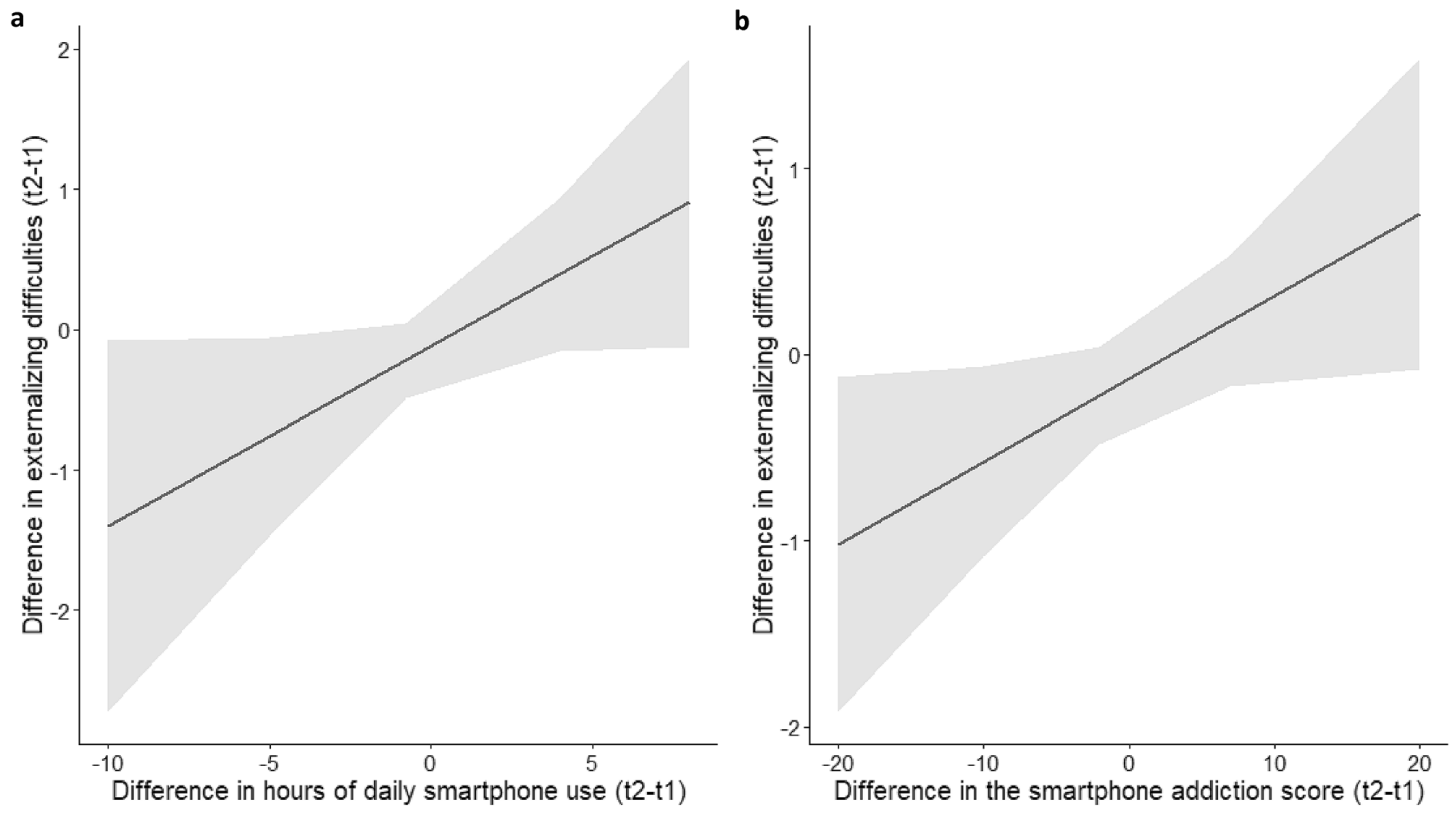
Effect plots illustrating the estimated change (+ 95% Confidence Interval) in externalizing behavioral difficulties within a period of 1 year depending on the change in the duration of daily smartphone use (**a**) and the change in symptoms of smartphone addiction (**b**) in the same period of time. The associations are adjusted for age, sex, and SES

The cross-sectional analyses revealed significant associations between levels of internalizing and externalizing difficulties on the one side and the duration of daily smartphone use and symptoms of addiction on the other (see Table 1). The longitudinal analyses indicated that increased levels of externalizing difficulties between t1 and t2 were significantly associated with an increase in the hours of daily smartphone use (*b* = 0.13 (95% CI 0.01–0.26), *p* = 0.049, see Fig. 1a) and an increase in symptoms of smartphone addiction (*b* = 0.04 (95% CI 0.00–0.09), *p* = 0.037, see Fig. 1b). Regarding changes in internalizing difficulties, the associations with, respectively, changes in the duration of daily smartphone use [*b* = 0.09 (95% CI − 0.05–0.23)] and with signs of smartphone addiction [*b* = 0.02 (95% CI − 0.02–0.07)] were also positive but not significant (*p* = 0.213 and 0.368, respectively).

**Table 1:** Significant positive cross-sectional associations (indicated by non-standardized regression coefficients + 95% Confidence Interval) between daily smartphone use/symptoms of smartphone addiction (independent variables) and externalizing and internalizing behavioral difficulties (dependent variables) at t1 and t2^a^

	Externalizing difficulties	Internalizing difficulties
t1		
Total smartphone addiction score	0.13 (0.08, 0.17)***	0.13 (0.08, 0.17)***
Hours daily smartphone use	0.18 (0.01, 0.35)*	0.25 (0.07, 0.43)**
t2		
Total smartphone addiction score	0.15 (0.11, 0.20)***	0.11 (0.06, 0.16)***
Hours daily smartphone use	0.38 (0.20, 0.55)***	0.36 (0.17, 0.56)***

^a^All associations are adjusted for age, gender, and SES

## Discussion

Our findings are indicative of a concurrent increase in externalizing difficulties and problematic smartphone use within a 12-month period. This strengthens the assumption of a long-term relationship between problematic smartphone use and externalizing difficulties, e.g., symptoms of inattention or aggression. One possible explanation for this finding is that adolescents with externalizing difficulties feel a need for immediate reward and responses, which is satisfied and might even be reinforced using a smartphone [7]. Another possible explanation is that both variables are influenced by a third factor such as impulsivity, dysfunctional regulatory strategies or inadequate executive functions. The findings are also clinically relevant. They indicate, for example, that in the case of externalizing behavioral problems, smartphone use should also be assessed or treated in parallel (and vice versa). With regard to internalizing difficulties (e.g., depressive symptoms, sadness, or loneliness), in contrast, the findings suggest that these may occur simultaneously with problematic smartphone use (as indicated by significant cross-sectional associations) but that, in the long term, the relationship is not one of mutual dependence.

One clear strength of the present study is its longitudinal design. However, we acknowledge that the underrepresentation of families from low social strata might limit the generalizability of the study findings to the general population of children and adolescents.

## Data Availability

Data cannot be shared publicly because there exist ethical restrictions. The LIFE Child study is a study collecting potentially sensitive information. Publishing data sets is not covered by the informed consent provided by the study participants. Furthermore, the data protection concept of LIFE requests that all (external as well as internal) researchers interested in accessing data sign a project agreement. Researchers that are interested in accessing and analyzing data collected in the LIFE Child study may contact the data use and access committee (forschungsdaten@medizin.uni-leipzig.de).
